# Development of a brief menstrual quality of life measure for women with heavy menstrual bleeding

**DOI:** 10.1186/s12905-023-02235-0

**Published:** 2023-03-14

**Authors:** Deborah Lancastle, Helena Kopp Kallner, Gabrielle Hale, Bethan Wood, Lauren Ashcroft, Holly Driscoll

**Affiliations:** 1 University of South Wales, Treforest Campus, Pontypridd, CF31 9DL Wales, UK; 2 Department of Clinical Sciences at Daneryd Hospital, Karolinska Insitutet, Stockholm, Sweden

**Keywords:** Heavy menstrual bleeding, Menstruation, Menorrhagia, Quality of life, Help-seeking, Decision making, PERIOD-QOL

## Abstract

**Background:**

The National Institute for Health and Care Excellence advises that considerations around quality of life should be made when assessing and treating heavy menstrual bleeding. A quick and reliable method for women to assess the impact of HMB on their quality of life might encourage help-seeking. This research aimed to develop a new 10-item measure of menstrual quality of life (the PERIOD-QOL).

**Methods:**

Three pilot studies describe PERIOD-QOL development and a cross-sectional survey (N = 376) assessed PERIOD-QOL scores in women who reported HMB and those who did not. A population sample of women (mean age 30.29, SD = 9.06) completed the PERIOD-QOL and rated their menstrual bleeding as heavy/very heavy/extremely heavy (HMB group) or very light/light/moderate bleeding (LMMB) group. Data were analysed using independent samples Analysis of Variance and independent samples t-tests.

**Results:**

Cronbach’s Alpha for the PERIOD-QOL = .88. A significant reduction in PERIOD-QOL scores was found across the 6 levels of bleeding from very light to extremely heavy, and significantly lower PERIOD-QOL scores were reported in the HMB than the LMMB group.

**Conclusion:**

The results suggest that the PERIOD-QOL is a reliable measure and that women experiencing HMB reported significantly lower menstrual quality of life than those who did not. Further validation of the PERIOD-QOL is required to determine its relationships with existing measures of menstrual quality of life and to establish whether PERIOD-QOL scores are associated with decisions to seek help from health professionals and with verified diagnoses of conditions that cause HMB.

## Background

Despite heavy menstrual bleeding causing substantial disruption to women’s lives [1], women can be reluctant to seek medical help unless attempts to self-manage their symptoms fail [2]. Women were worried that their symptoms were too “trivial” ([2], p.282) to consult a doctor about, and that people might think they were making an unnecessary fuss about a natural part of womanhood. A reliable tool that women can use to evaluate the impact of HMB on their lives could alert them to the impact of their heavy periods and help them make decisions about seeking help.

Women’s efforts to normalise and self-manage HMB can reduce the likelihood of them seeking help for gynaecological conditions causing HMB. For example, uterine fibroids can cause heavy bleeding and pain, as well as disruption to work attendance and performance, care of families, sport and leisure activities, holidays, clothing choice, and psychological wellbeing [3–6], but it can take women up to 5 years to receive help for this condition [7]. Understanding delays in seeking and receiving treatment for HMB is important. Some women in the study by Santer et al. ([2], p.282) said their doctors had been “dismissive” of their symptoms, which could contribute to delays in receiving help. However, another potential issue could be women’s difficulty in gauging whether their bleeding is abnormally heavy. Although HMB is defined as loss exceeding 80 ml per menstrual cycle [8, 9], this is difficult for women to measure. There are several methods of assessing blood loss during menstruation, including measuring the blood content of used sanitary products [10], assessing the iron content of menstrual loss [11], using collection devices such as the ‘Mooncup’ [12], or using pictorial charts which assess the number of sanitary products used and the amount of loss in each [13]. However, such methods can be impractical in a home or clinical setting [14]. In addition, developments in sanitary products affect absorbency of products and frequency of changes [14], the content of collection devices can be spilled and lost [12], and it would be difficult to assess any leaked blood that is not contained within sanitary products [14]. The National Institute of Care Excellence [NICE] guidelines (NG88, [15]) recommend that health professionals’ assessments of menstrual bleeding should include discussions with the woman about whether she believes her menstrual bleeding falls outside of the normal range of severity. This approach relies on women’s self-perceptions of HMB rather than objective measurements of blood loss with their various limitations, but is itself limited because women might be “poor judges” of blood loss. It also requires them to have observed and recorded or remembered their periods ([14], p. 8).

However, NICE also recommends that health professionals enquire about the impact of HMB on women’s quality of life and advise that interventions should focus on improving quality of life as well as blood loss. Quality of life is a complex construct involving a person’s perception of their physical, psychological, and social wellbeing and is often assessed in health contexts where health conditions are known to affect various biopsychosocial outcomes of importance to patients. Given the emphasis NICE places on the impact of HMB on quality of life, it is important that assessment of menstrual quality of life is reliable and valid [16]. Measures of general health-related quality of life have been used in the context of menstrual disruption (e.g., the Short-Form [SF-36] Health Survey, [17, 18]) and there are also measures specific to menstrual QOL such as the 24 item multiattribute utility assessment for menorrhagia [19], and the 20-item Menstrual Bleeding Questionnaire [20]. Measures containing 20–30+ items are likely to be fairly time consuming to complete, particularly for those with low literacy or who are not familiar with the English language, although recent work has addressed time burden with brief measures of menstrual quality of life (e.g., the six-item SAMANTA questionnaire and a two item visual analogue scale, [21, 22]. However, the measures above were developed for use in clinical practice and thus are more likely to be completed by women who have already consulted a health professional. Given that women can delay seeking help for HMB, a quick and reliable self-administered measure of menstrual quality of life for women to use could help women to monitor their periods, to evaluate the impact they have on their lives, and inform their decisions about seeking help. Moreover, the measure could provide a metric for health professionals to assess women’s experiences, and could facilitate assessment of treatment effectiveness. The present study describes the development of a new, brief measure of period-related quality of life (the PERIOD-QOL) designed for women in the general population to assess the physical, psychological, and social impact of periods on their lives.

## Methods

**Fig. 1:**
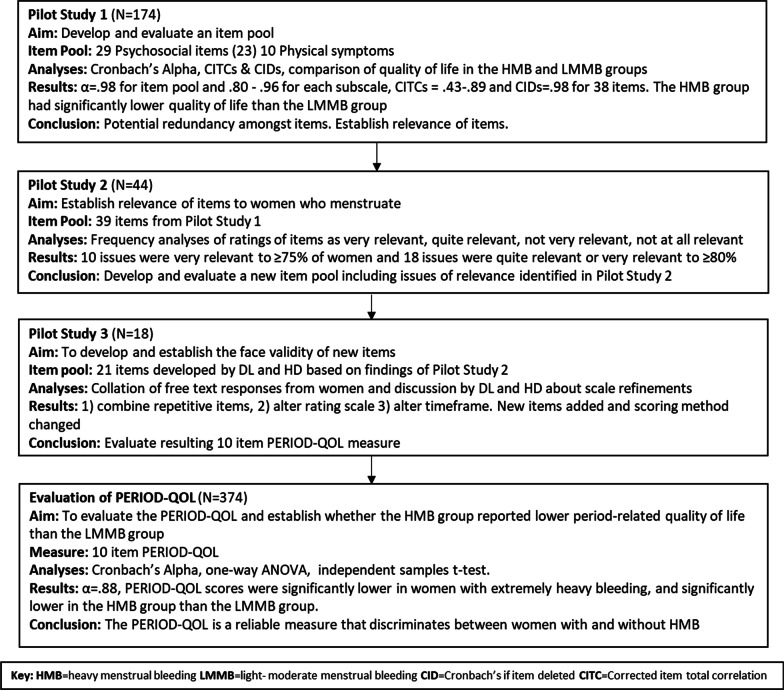
Flow chart showing process of PERIOD-QOL development

Figure 1 illustrates the process of PERIOD-QOL development.

*Aims* The aim of the present research was to develop a new, brief self-administered measure of menstrual quality of life (the PERIOD-QOL). A secondary aim was to establish whether women who experienced HMB reported lower scores on the PERIOD-QOL than women who did not.

*Hypotheses* It was expected that women reporting heavy menstrual bleeding would report significantly lower quality of life on the PERIOD-QOL than women who did not.

### Pilot study 1: generation and evaluation of item pool

We used a pool of 39 items enquiring about the biopsychosocial aspects of HMB. The 29 psychosocial items were from the Uterine Fibroid Symptom and Quality of Life scale (UFS-QOL; 23). These items enquired about period-related issues such as embarrassment, lack of control, mood problems and fatigue, disrupted activities etc., associated with uterine fibroids. We also developed 10 items enquiring about physical aspects of menstruation, including heavy bleeding, pain, missing periods, and intermenstrual bleeding.

#### Methods

Women were invited to participate via advertisements placed on social media, online forums, and on noticeboards in the first author’s university. An opportunity sample of women from the general population (N = 174) aged between 18 and 55 (Mean = 31.71, SD = 9.43) completed a paper or online survey in which they responded to the 39 items on 5 point scales ranging from ‘Not at all’ to ‘A very great deal’ (Physical symptoms) or ‘None of the time’ to ‘All of the time’ (psychosocial symptoms) by considering their experiences over the last three months. Scores for the 29 psychosocial items were transformed and six subscales were created from these items (see Table 1), in line with the method of Spies et al. [23]. Higher scores represented better quality of life in these domains. The Physical Symptoms subscale was reverse scored and summed, with higher scores equalling better physical quality of life. We also developed a total quality of life score from the sum of all subscale scores. See Table 1 for descriptive statistics and Cronbach’s alphas for these subscales. Women also completed a demographic and reproductive history questionnaire which enquired about age, ethnicity, and reproductive health. In response to the question “In your opinion, are your periods *usually*…”, women rated their usual menstrual bleeding on the following 6 point scale:

**Table:** 

Very light 1	Light 2	Normal/moderate 3	Heavy 4	Very heavy 5	Extremely heavy 6
I think I lose much less blood than other women	I think I lose a bit less blood than other women	I think I lose about the same amount of blood as other women	I think I lose a bit more blood than other women	I think I lose much more blood than other women	I think I lose so much more blood than other women that I am worried about it

Women who rated their bleeding as very light/light/moderate were assigned to the light or moderate bleeding (LMMB) group and those rating their bleeding as heavy, very heavy, or extremely heavy were assigned to the heavy menstrual bleeding (HMB) group.

## Results

Women in the HMB group reported significantly greater distress from their physical symptoms, lower QOL on all psychosocial subscales, and lower total quality of life than women in the LMMB group (see Table 1).

**Table 1:** Differences in menstrual quality of life scores between women in the HMB and LMMB groups

Subscale	Number of items (α)	HMB group (n = 115) mean (SD)	LMMB group (n = 59) mean (SD)	t (172)	d
Physical symptoms	10 items α = .89	59.53 (22.22)	29.97 (18.42)	− 8.78***	− 1.41
Energy and mood	7 items α = .96	26.06 (26.55)	58.73 (31.33)	7.22***	1.16
Control	4 items α = .92	30.87 (29.74)	74.08 (26.14)	9.44***	1.50
Activities	6 items α = .96	36.99 (30.83)	75.71 (27.62)	8.12***	1.30
Self-consciousness	3 items α = .80	40.29 (28.72)	73.03 (24.73)	7.45***	1.19
Concern	7 items α = .91	38.73 (26.25)	76.40 (23.30)	9.33***	1.50
Sexual function	2 items α = .83	26.96 (29.97)	59.11 (33.06)	6.47***	1.04
Total QOL	39items α = .93	33.58 (25.28)	70.13 (23.93)	9.19***	1.47

***p < .001

The results showed that these subscales were reliable and discriminated between women who did or did not report HMB. Further examination of data showed that the 39-item pool yielded a Cronbach’s Alpha of 0.98. Corrected Item total correlations (CITCs) and Cronbach’s if Item Deleted values (CIDs) were examined to determine if there were any items with CITCs < 0.3. One item ‘Missing periods’ (from the Physical Symptoms subscale) had a CITC of 0.16 and CID of 0.98. The CITCs of all other items ranged between 0.43 for bleeding between periods on the Physical Symptoms subscale to 0.89 for items enquiring about daily routine and not achieving plans, and CIDs for all items were 0.98. Clark [16] emphasised that as well as having good measurement properties, developers of quality of life measures should ensure that their measures assess the problems that are important to people living with a particular condition. Therefore, in Pilot Study 2, we evaluated the face validity of the issues described in the item pool in terms of the relevance of the issues to women’s menstrual quality of life [16].

### Pilot study 2: relevance of the item pool

The aim of Pilot Study 2 was to determine the relevance of the issues represented in the item pool to women who menstruate.

#### Methods

Women in the general population were invited to participate via a link to an online survey posted on social media websites by LA. The final sample included 44 women aged between 18 and 52 (mean = 29.67, SD = 9.79), all of whom experienced menstrual periods. Nearly all women described themselves as white (95.5%, n = 42) and there were two Asian women. The majority had English as a first language (88.8%, n = 39), and four others said they were equally proficient in English and in other languages. Nearly two-thirds (63.64%, n = 28) had undergraduate degrees, three (6.8%) had secondary education qualifications, seven (15.9%) had advanced secondary education or vocational qualifications, and nine (20.5%) had higher qualifications. In response to the question about severity of menstrual bleeding (as used in Pilot Study 1), four (9.1%) of women had light periods, 22 (50%) women’s periods were normal/moderate, six women’s (13.6%) were heavy, eight women’s (18.2%) were very heavy, and four women (9.1%) had extremely heavy periods. Women were asked about their experience of period pain and four said they did not experience pain in their last period, six had light pain, 21 had moderate pain, nine had severe pain, and four had unbearable pain. In response to a question asking whether they had ever consulted a doctor about period problems, five had never had period problems, 22 had never consulted a doctor, and 17 had consulted a doctor. Four women had diagnoses of endometriosis and/or fibroids, and one woman had a diagnosis of menorrhagia and anaemia. The final sample was taken from an initial sample of 54 women. Ten were excluded because they did not menstruate, due to menopause (n = 4), contraception (n = 2), hysterectomy (n = 3) and other medical treatment (n = 1).

Women were provided with the item pool and were asked to rate the relevance of each physical, psychological, and social effects of period problems according to whether they considered it to be very relevant, quite relevant, not very relevant, or not at all relevant. Frequency analyses showed that the issues women would consider very relevant were: cramps and pain (86.4%), premenstrual syndrome (77.3%), and bleeding that was heavy, unexpected, that happened between periods, or lasted longer than seven days (all 75%). The psychosocial issues considered very relevant were feeling irritable, (81.8%) feeling self-conscious about sanitary protection (70.5%), unpredictable periods, and feeling sad or hopeless (all 75%). Nearly all women considered issues with their energy or mood such as tiredness, weakness, or negative mood to be very relevant or quite relevant (92.2–97.7%). Other psychosocial issues considered very relevant or quite relevant by > 4 out of 5 women were the impact of periods on travelling (86.54%), physical activities (90.9%), exercise (84.1%), daily routine (88.6%), social life (84.1%), sexual desire and sexual relations (88.6% and 90.9%, respectively). Women also thought soiling of underwear (86.4%) or bedding and furniture (88.6%), carrying sanitary protection (88.6%), stomach bloating (95.5%), health worries (86.4%) and uncertainty (84.1%) were very relevant or quite relevant. Finally, most women considered problems with control over their lives (84.1%) and bodies (95.5%), plans (89.6%), achievement (84.1%) and clothing choices (88.6%) to be very relevant or quite relevant. These results confirmed the relevance of numerous issues in the item pool to women’s menstrual quality of life. We therefore proceeded to develop a new, smaller item pool which contained physical health issues considered very relevant by women (bleeding, cramps/pain, premenstrual syndrome and unexpected, irregular, lengthy, and intermenstrual bleeding). In terms of psychosocial issues, we developed two items about tiredness and fatigue, two about control over clothing and life, three about irritability, moods, and mental health, two about period-related effects on self-consciousness, and one item each about confidence, soiling, health worries, sex life and disruption to activities. Finally, we added in two items about the impact of periods on work or school (attendance and performance). These revisions yielded a pool of 21 items. We then conducted another pilot study to determine women’s opinions about these items and the instructions and rating scale.

### Pilot study 3. relevance of the new item pool

We invited 18 women aged 21–39 (mean = 26.44, SD = 6.48) known to HD to review (1) these 21 items, (2) the rating scales (3) the three month timeframe for consideration of menstrual issues. Due to restrictions on face to face data collection during the Covid 19 pandemic, data collection was managed via email. Seventeen of the participants described their ethnicity as white (one was Asian). Five had secondary education qualifications, one had advanced secondary education qualifications, nine had undergraduate degrees, and three had higher qualifications. All women menstruated, and three reported experiencing HMB. Participants were sent the pool of 21 items and they provided text comments about the items, in response to the following criteria:

1. Which items they did/not think were relevant to period-related quality of life
2. Their opinions about the rating scale
3. Whether anything was missing that should be added
4. Whether any of the questions were confusing or hard to read/understand
5. Whether they had any general feedback

Women’s responses were collated by HD, and DL and HD discussed women’s responses and made the following changes based on their feedback: (1) The number of months women should consider when evaluating menstrual issues was altered from 3 to 6 months because this better reflected consistent changes or problems, (2) We changed 1–5 rating scales to options enquiring about the number of months in which a problem was present: Never, Once or twice, A few times (2–3 periods), In most periods (4–5 periods) and In every period, because this would make problems easier to quantify. Questions enquiring about premenstrual syndrome, body consciousness, self-consciousness, and self-confidence were removed because they were not relevant to the problems women said they experienced during menstruation. Women also suggested that we combine separate items assessing unexpected bleeding and irregular periods, as well as combining questions about fatigue and about mood. We developed a new item asking about severe period pain to better assess the nature of pain that impacted on women’s quality of life, and we included embarrassment in a question about leaking and soiling of clothes. Finally, item scoring was changed so that it ranged from 5 (never) to 1 (in every period) to avoid the need for women to transform or reverse score items, thus increasing ease of self-administration (i.e., feasibility, 16). Higher scores equalled better quality of life. Together, this feedback resulted in a 10-item measure, which we called the PERIOD-QOL (see Table 2).

**Table 2:** The PERIOD-QOL

How have your periods been over the last 6 months? Please tick one box for each question					
My periods make me bothered or upset because…	Never (5)	Once or twice (4)	A few times (2–3 periods) (3)	In most periods (4–5 periods) (2)	In every period (1)
They are extremely heavy					
I have severe period pain					
I bleed unexpectedly, between periods or for more than 7 days					
I am worried or embarrassed about leaking and soiling clothes or bedding					
They make me worry about my health					
They interfere with exercise, clothing choices, travel, holidays, or socialising					
They make me miss time at work/school or my periods affect my work while I am there					
They make me feel tired or drained					
They make me feel moody, sad, irritable, or fed up					
They affect my sex life					

### Evaluation of the PERIOD-QOL

#### Design

The study was of independent samples design. The non-manipulated independent variable was severity of menstrual bleeding. The dependent variable was PERIOD-QOL score.

#### Participants

The final sample included 376 women aged between 18 and 53 (mean = 30.21, SD = 8.81). This was an opportunity sample who were recruited from the general population via adverts on social media and online forums such as FibroidsConnect. All menstruating women were eligible to participate, regardless of whether they experienced HMB. Almost all women described their ethnicity as White (n = 358, 95.2%), eight (2.1%) were Asian, 8 (2.1%) were of mixed or other ethnic groups and less than 1% (n = 3) were Black. More than half of the women were employed (n = 207, 55.1%) and over one-third were university students (n = 137, 36.4%). The final sample was recruited from 382 women who consented to participate in the study. Six women (1.6%) were excluded because they were younger than 18 years, which was the minimum age for participation sanctioned by the ethics panel. Participants were volunteers and there was no remuneration for participating.

#### Materials

The survey was presented via JISC Online Surveys (https://jisc.onlinesurveys.ac.uk) and included the following measures:

*Demographic and reproductive history questionnaire* This questionnaire enquired about women’s age, ethnicity, and reproductive health. The assessment of the severity of menstrual bleeding and assignment to LMMB or HMB groups followed the methods used in Pilot Studies 1 and 2.

*The PERIOD-QOL* Women completed the 10-item PERIOD-QOL by reflecting on their experiences of how ‘bothered and upset’ they had felt about their periods over the last 6 months. Principal Components Analysis of the PERIOD-QOL yielded one 10 item scale accounting for 47.9% of the variance in quality of life (α = 0.88). Scores on the PERIOD-QOL were therefore summed, with higher scores representing better quality of life.

#### Procedure

Data were collected between February and June 2021. Women were recruited from advertisements on social media sites (Facebook, twitter, Instagram) and on online forums for women with gynaecological problems (e.g., Fibroidsconnect). Women were invited to participate regardless of whether they experienced any menstrual problems. On submitting the survey, women received an online debrief form and were thanked for participating. Suggestions about sources of help and support were provided, and women were advised to contact their own doctors with any worries about their physical or psychological wellbeing.

#### Data analysis

The required sample size of 64 participants per group for independent samples t-test analysis was determined by G*power analysis [24] for a medium effect size (Cohen’s d = 0.5), statistical power 0.80 and probability of < 0.05, two tailed. Quantitative analyses were conducted using the statistical software packages SPSS [25]. Data were screened for normality. The skewness coefficients for the PERIOD-QOL (SE = 0.13) was 0.06 and the coefficient for kurtosis (S.E = 0.25) was − 2.5. A one-way ANOVA with Tukey posthoc analysis was conducted to establish differences in PERIOD-QOL across the six levels of menstrual bleeding severity. Independent samples t-tests were conducted to determine differences in PERIOD-QOL scores in women in the LMMB and HMB groups. The significance level accepted was *p* < 0.05.

#### Results

There were equal numbers of women in the LMMB group and HMB groups (see Table 3).

**Table 3:** Frequencies (number, percentage) of women reporting each level of bleeding severity

LLMB group (n = 188, 50%)			HMB group (n = 188, 50%)		
Very light	Light	Normal/moderate	Heavy	Very heavy	Extremely heavy
37 (9.8%)	39 (10.4%)	112 (29.8%)	106 (28.2%)	60 (16.0%)	22 (5.9%)

**Fig. 2:**
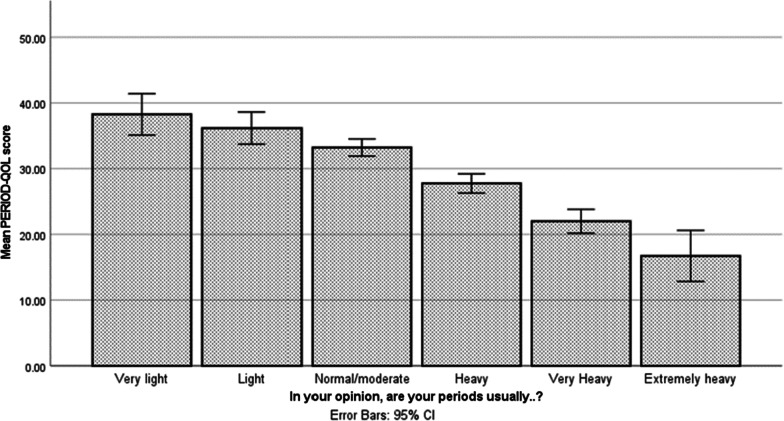
Mean PERIOD-QOL scores at each level of menstrual bleeding severity

The mean PERIOD-QOL score for the whole sample was 29.73 (SD = 9.63), and scores ranged from 10 to 50. A significant difference in PERIOD-QOL scores was found across the 6 levels of bleeding severity, F (5, 370) = 46.43, *p* < 0.001, η2 = 0.39 (see Fig. 2). Women who reported extremely heavy bleeding experienced significantly lower quality of life than those who reported any other level of bleeding severity (all *p* < 0.05). The mean PERIOD-QOL score for women in the HMB group (24.63, SD = 8.47) was significantly lower than that in the LMMB group (34.83, SD = 7.86), t (374) = 12.10, *p* < 0.001, d = 1.25.

## Discussion

The present research presents the development of the PERIOD-QOL, which is a new 10-item measure of the impact of menstruation on women’s quality of life We conducted a number of studies during development of the PERIOD-QOL, and our findings suggest that the PERIOD-QOL has good internal consistency and that scores for women who reported HMB were significantly lower than scores of women who did not. This group difference was expected given the impact of HMB on quality of life demonstrated in numerous studies, therefore indicating that the PERIOD-QOL has construct validity. Our development of the PERIOD-QOL included seeking the opinions of women about the relevance of various menstrual issues, thus helping to confirm the face validity of the measure. Together, these studies satisfy some of the measurement properties and face validity criteria for quality assessment of menstrual quality of life measures proposed by Clark and colleagues [16]. Further research is needed to demonstrate other measurement properties of the PERIOD-QOL by validating it against existing measures of menstrual QOL, and amongst women with verified HMB and diagnoses of conditions causing HMB. The PERIOD-QOL is designed for women in the general population to self-assess their periods. Developing the measure into a self-help tool which includes guidance on seeking help for HMB after completing the PERIOD-QOL could be provided via an application or an internet site. This would increase the accessibility of the measure and provide a tool that could help women to monitor the impact of their periods on their quality of life and encourage them to seek medical help.

The provision of aids to support women in decisions to seek assessment and treatment for menstrual problems is important to minimise delays in receiving help for any medical problem that might be affecting menstruation. It is important that any such aid is understandable to women with lower levels of education or literacy, who may struggle to understand unfamiliar information and complex vocabulary [26]. We endeavoured to ensure PERIOD-QOL items were relevant by developing the measure in consultation with women and refining items in line with their feedback. This process helped to confirm the face validity of the items [16]. In addition, women of different educational backgrounds, ranging from secondary education qualifications to higher degrees contributed to the development of items, which should increase the likelihood that the PERIOD-QOL can be understood by women of various educational backgrounds. However, few women of different ethnic backgrounds contributed to the development of the PERIOD-QOL. Further research is needed to establish whether items are understandable and relevant to those with different ethnic or language backgrounds. Moreover, further research is needed establish the convergent validity of the PERIOD-QOL [16] by examining its relationships with existing measures of menstrual quality of life [19–22], and its predictive validity via the extent to which its use predicts relevant outcomes, such as help-seeking for HMB. Should future research confirm its reliability and validity the PERIOD-QOL could also prove to be useful in primary and secondary health care settings. Finally, if women complete the PERIOD-QOL in advance of consulting a health professional it could enhance patient-practitioner communication by providing women with greater awareness of the nature of the disruption caused by their periods and a score that provides a metric for assessing the severity of that disruption.

### Limitations

One limitation of the present research is that participants contributed anonymously and the severity of their menstrual bleeding has not been confirmed by medical records. We also did not enquire about factors that can influence menstrual flow such as menopausal status or contraception. Many perimenopausal women consult health professionals about abnormal bleeding, including heavy menstrual bleeding and bleeding between periods [27] and the age range of our sample would suggest that at least some women contributing to this study were perimenopausal or menopausal. In addition, some contraceptive methods can impact menstrual flow, with around two-thirds of women with a copper intrauterine contraceptive device experiencing heavy periods [28]. However, the aim of the present study was to develop the PERIOD-QOL as a tool that could assess the quality of life implications of HMB, regardless of its cause. If menopause or contraceptive methods are causing quality of life limiting problems with menstruation, consulting a health professional for help would still be appropriate. Future research should continue to validate the tool and determine its utility in assessing period-related quality of life for specific groups of women (e.g., adolescents, (peri)menopausal women and those using different forms of contraception). In addition, prospective study designs would be appropriate to determine the causal relationships between period-related quality of life, help-seeking, diagnoses, and other indices of wellbeing.

## Conclusion

The aim of the present research was to develop a brief measure of period-related quality of life and to establish whether women who reported HMB would report lower quality of life on this measure than women who did not. The results demonstrated that PERIOD-QOL was reliable, and that scores were significantly higher in women with self-reported heavy menstrual bleeding compared to those who do not experience this issue. The PERIOD-QOL therefore seems to have potential utility as a self-administered measure for women to use to assess the impact of HMB on their quality of life. Further validation of the PERIOD-QOL and development of the measure into an application or online tool with accompanying guidance could increase the accessibility of the measure and encourage women to seek assessment and treatment for quality of life limiting HMB.

## Data Availability

The datasets used and/or analysed during the current study are available from the corresponding author on reasonable request.
